# Alkyl-Capped Silicon Nanocrystals Lack Cytotoxicity and have Enhanced Intracellular Accumulation in Malignant Cells via Cholesterol-Dependent Endocytosis

**DOI:** 10.1002/smll.200800903

**Published:** 2008-12-04

**Authors:** Naif H Alsharif, Christine E M Berger, Satya S Varanasi, Yimin Chao, Benjamin R Horrocks, Harish K Datta

**Affiliations:** School of Natural Sciences, Bedson Building Newcastle UniversityNewcastle upon Tyne NE1 7RU (UK); Musculoskeletal Research Group, Institute of Cellular Medicine Newcastle UniversityFramlington Place Newcastle upon Tyne, NE2 4HH (UK); A & R Biology Pfizer Limited500/1.826 IPC 675 Ramsgate Road Sandwich CT13 9NJ (UK); Biomedical Tissue Research Group Department of Biology University of York YorkYO10 5DD (UK); Faculty of Science Chemical Sciences and Pharmacy University of East AngliaNorwich NR4 7TJ (UK)

**Keywords:** cells, fluorescence, nanoparticles, quantum dots, silicon

## Abstract

Nanocrystals of various inorganic materials are being considered for application in the life sciences as fluorescent labels and for such therapeutic applications as drug delivery or targeted cell destruction. The potential applications of the nanoparticles are critically compromised due to the well-documented toxicity and lack of understanding about the mechanisms involved in the intracellular internalization. Here intracellular internalization and toxicity of alkyl-capped silicon nanocrystals in human neoplastic and normal primary cells is reported. The capped nanocrystals lack cytotoxicity, and there is a marked difference in the rate and extent of intracellular accumulation of the nanoparticles between human cancerous and non-cancerous primary cells, the rate and extent being higher in the malignant cells compared to normal human primary cells. The exposure of the cells to the alkyl-capped nanocrystals demonstrates no evidence of in vitro cytotoxicity when assessed by cell morphology, apoptosis, and cell viability assays. The internalization of the nanocrystals by Hela and SW1353 cells is almost completely blocked by the pinocytosis inhibitors filipin, cytochalasin B, and actinomycin D. The internalization process is not associated with any surface change in the nanoparticles, as their luminescence spectrum is unaltered upon transport into the cytosol. The observed dramatic difference in the rate and extent of internalization of the nanocrystals between malignant and non-malignant cells therefore offers potential application in the management of human neoplastic conditions.

## Introduction

Nanoparticles have many applications in life sciences,^1^–^3^ but there is immense concern relating to their potential toxicological hazard to the environment.^4^–^6^ To mitigate this concern, alkyl-capped silicon nanocrystals (alkyl-SiNCs) have been suggested as useful nontoxic, red-emitting fluorescent labels.^7^,^8^ Semiconductor nanocrystals (NCs), also known as quantum dots (QDs), are of immense interest for medical imaging because of their controllable photophysical properties.^9^–^11^ NC-antibody conjugates can be designed to target specific cell types and tissues and allow therapeutic agents to be delivered to the affected cells and tissues.^12^ It is envisaged that by employing NCs medical diagnostic imaging can be extended beyond merely visualizing gross pathology and cell morphology.^1^ NCs are being used to carry out cellular localization of nucleic acids, proteins, and other macromolecules, with the goal of detecting and correcting aberrant disease-related changes in expression.^13^,^14^ In addition to detecting and localizing molecular defects in gene and protein function, NCs have potential application for correcting cell-specific molecular defects.^15^ These applications require a thorough understanding of mechanisms involved in the cellular uptake, and intracellular and subcellular localization of the various types of nanoparticles.

The range and extent of nanoparticle applications is expected to rise, thereby increasing the risk of unwanted exposure to humans as well as the environment.^16^ There is therefore an urgent need to understand the mechanisms of potentially harmful side effects of these materials, with the view that this understanding will allow the manufacture of NCs with minimal hazardous effects and informed risk assessment. The best understood and most widely used quantum dot fluorophores are based on cadmium chalcogenide NCs.^17^ However, in vitro studies suggest that under certain conditions this type of nanoparticle may be cytotoxic. This cytotoxicity is primarily attributed to the leaching of heavy metal ions such as Cd^2+^, but some reports indicate the formation of free radicals.^4^,^6^ To address this problem we and others have synthesized alkyl-capped silicon nanocrystals (alkyl-SiNCs) that are chemically stable and strongly luminescent.^7^–^9^ In order to establish if alkyl-SiNCs could be employed to study intracellular functions without disrupting activity, we carried out in vitro cellular studies using primary and transformed cell-lines of human origin. These studies were designed to determine the possible toxicity and mechanisms involved in cellular internalization of alkyl-SiNCs. These intensely fluorescent alkyl-SiNCs, while showing no evidence of in vitro cytotoxicity, accumulated within the cytosols of human malignant and normal primary cells at markedly different rates.

## Results

### Physical Characteristics of SiNCs

**Figure 1:**
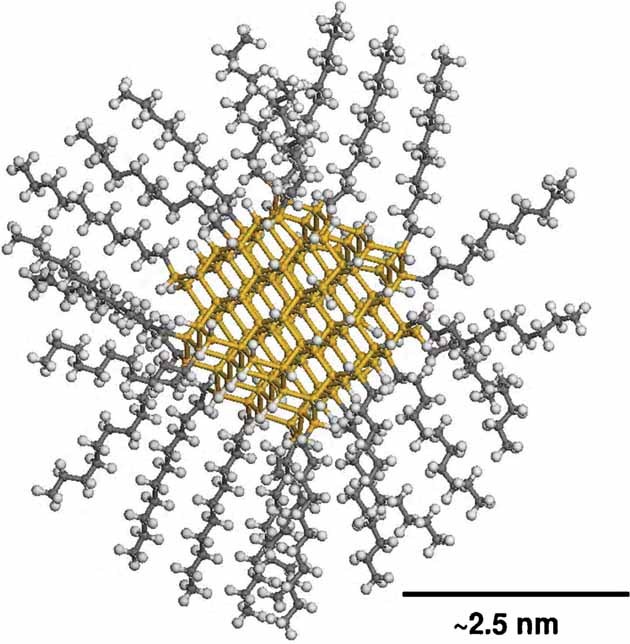
Schematic molecular model of the SiNCs used in this work. The luminescent silicon core is crystalline with similar lattice parameters to the bulk. The diameter of the core is about 2.5 nm and the diameter of the whole particle including the undecyl capping layer is about 5 nm.

The NCs comprise a crystalline silicon core covered by a C_11_ alkyl monolayer (Figure 1). The silicon core is 2.5 nm in diameter and the size of the whole particle, including the alkyl monolayer, is about 5 nm (detailed characterization is provided in References 18,20,21). The NCs are intensely luminescent under excitation with *λ*_ex_ < 550 nm and the emission spectrum is independent of the excitation wavelength.^21^ The NCs are highly hydrophobic and therefore have negligible miscibility in water. They are very soluble in nonpolar solvents, and solutions thereof can be mixed with excess water to form a lyophobic sol that is stable against flocculation for periods up to several months.^20^ A range of solvents were used, including tetrahydrofuran (THF), toluene, dimethylsulfoxide (DMSO), and diethyl ether (ether). The NCs were found to be most readily soluble in THF, toluene, and ether.

### In vitro Cytotoxicity Assays

**Figure 2:**
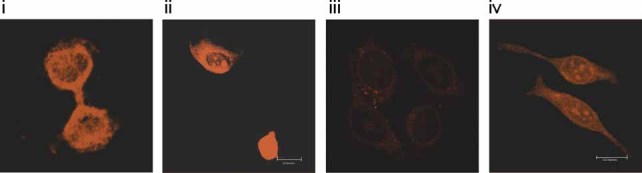
Images from screening carried out to select suitable solvents for making alkyl-SiNC suspensions for use in the in vitro studies with Hela cells cultured in monolayer. Confocal laser fluorescent images, obtained at *λ*_excitation_ 488nm and *λ*_emission_ between 550 and 650 nm, of cells grown in 1 mL of DMEM and 10% FCS and exposed for 1 h to alkyl-SiNCs suspended in 2 µL THF (i), toluene (ii), DMSO (iii), and ether (iv).

The screening to detect the most effective solvent was performed using Hela cells and freshly isolated primary human skin fibroblasts (HSFs). In this screening procedure, morphological features of cell necrosis and plasma membrane blebbing were observed. These studies demonstrated that alkyl-SiNCs suspended in THF, toluene, DMSO, and ether were avidly taken up by the Hela cells (Figure 2). However, THF and toluene were found to be acutely cytotoxic even at the low volume fractions (0.1%) required to disperse the alkyl-SiNCs in water. The cells were found to show blebbing and acute cell necrosis within 30 min of exposure to the alkyl-SiNC suspensions in these solvents or to the respective vehicle alone. In contrast, alkyl-SiNCs suspended in DMSO and ether showed no evidence of acute cytotoxicity despite a significant spontaneous uptake of alkyl-SiNCs from these suspensions. The comparison of ether and DMSO showed a markedly higher rate of alkyl-SiNC uptake, as well as peak accumulation of alkyl-SiNCs, when using ether suspensions as compared to DMSO. Further, alkyl-SiNCs showed lower miscibility in DMSO than in ether, requiring a prolonged period of incubation.

**Figure 3:**
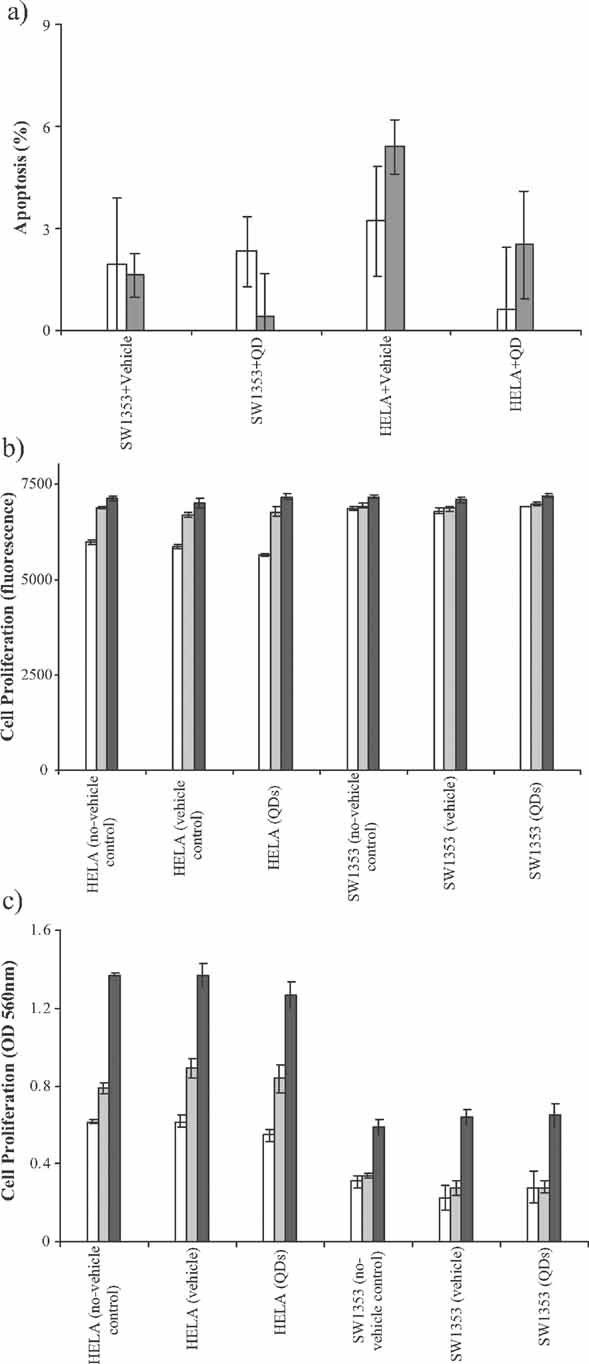
Screening for alkyl-SiNC cytotoxicity. a) Hela and SW1353 cells were exposed to 2 µL of nanocrystals or vehicle in 1 mL culture medium for 30 min (white columns), and 1 h (dark columns), and observed under a phase-contrast microscope. There was no evidence of acute cytotoxicity, as evident from apoptosis (colorimetric TUNEL) assay performed on SW1353 and Hela cell-lines. b) The possible cytotoxic effect of NCs was excluded by the lack of effect in the cell proliferation and viability assays. SW1353 and Hela cells that had been starved overnight were exposed to medium containing dots or vehicle for variable times (1 h (white column), 4 h (gray column), and 24 h (dark column)), and cell viability assays were carried out using the CellTiter-Blue Cell Viability Assay. c) For the MTT assay, cells were exposed for to NCs for 2 h (white column), 4 h (gray column), and 24 h (dark column). The cells were exposed to either NCs suspended in 2 µL ether and controls were exposed to vehicle (2 µL ether) alone. All experiments ranged between *n* = 5 to 8 and were repeated on at least three different occasions.

In vitro cytotoxicity investigations, comprising apoptosis, cell viability, and proliferation assays, were carried out on Hela and SW1353 cells (human chondrosarcoma). In all these studies, solutions of alkyl-SiNCs in ether or vehicle alone (2 µL) were added to a Hela cell monolayer culture in 1 mL of culture medium for a variable period ranging between 1 h and 24 h. When the cells were observed under a phase contrast microscope there was no evidence of acute cytotoxicity, of cell necrosis, or of plasma membrane blebbing. The lack of observed cellular toxicity was further confirmed by the apoptosis assay (colorimetric TdT-mediated dUTP Nick-End Labeling (TUNEL) assay) performed on Hela and SW1353 cell-lines (Figure 3a). There were no significant differences in the rate of apoptosis between cells exposed to alkyl-SiNCs and those exposed to vehicle alone. A possible chronic cytotoxic effect of alkyl-SiNCs was investigated by exposing cells to alkyl-SiNCs for up to 24 h. These assays determined changes in the rate of cell proliferation and cell viability in the presence of constant exposure to alkyl-SiNCs in the culture medium (Figure 3b). It was found to show no effect on the rate of cell proliferation (the number of NCs in the medium was ∼3 × 10^12^ or 5 pmol). No effect on cell proliferation was seen even after cells had been exposed to alkyl-SiNCs for 24 h (Figure 3c).

### Kinetics of Internalization in Normal and Neoplastic Cells

**Figure 4:**
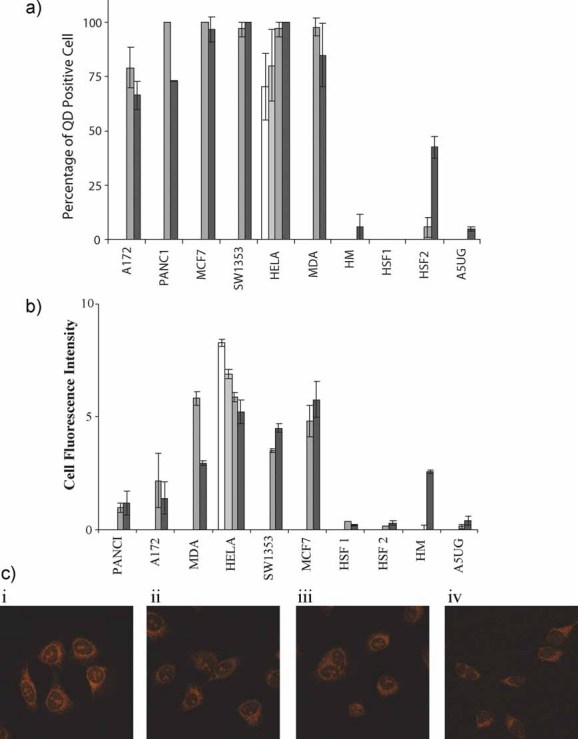
Human cell-lines and primary cells were incubated with NCs for variable times (30 min (white columns), 1 h (white gray columns), 2 h (dark gray columns), and 4 h (dark columns). After incubation the cells were washed twice with PBS and fixed with a fixative solution of 0.15 m citrate aqueous solution (40%) and acetone (60%) for 30 s. The fluorescence was determined using a laser confocal microscope (*λ*_excitation_ 488 nm and *λ*_emission_ between 550 and 650 nm). a) The quantitative analysis and comparison was carried out by determining the uptake, in terms of % positive cells and their fluorescence intensity, in six different fields on each slide. b) The fluorescence intensities were measured against control cells that had been exposed to an appropriate vehicle for same length of time as the NCs. In order to quantify the extent of intracellular NC accumulation, the NC fluorescence was determined (ranged between 5 to 8 different cells) using the Simulator software. c) Images of the representative fluorescent Hela cells at i) 30 min, ii) 1 h, iii) 2 h, and iv) 4 h. The orange color-scale approximately matches the luminescence.

Further studies, unless otherwise stated, were performed with alkyl-SiNCs suspended in ether, and in all studies the same concentrations of nanocrystals or vehicles alone were used. These investigations were performed on neoplastic cells of human origin, namely Hela (immortalized epithelial cervical carcinoma), A172 (glioblastoma tumor), MCF7 (breast adenocarcinoma), PANC1 (pancreatic epithelial-like carcinoma), SW1353 (chondrosarcoma), and MDA231 (breast cancer). Non-malignant primary human cells used were skin fibroblast (HSF1 and HSF2) cells that had been isolated from two different healthy subjects, and Human Myoblast (HM) and renal cells (A5UG). The uptake of the nanocrystals from the culture medium by cells, determined using laser confocal fluorescence microscopy, was estimated in terms of percentage of cells showing evidence of internalization as well as degree of intracellular accumulation. The study revealed spontaneous internalization of alkyl-SiNCs by both malignant and non-malignant cells, requiring the presence of albumin but without requiring any transfection procedure. Whilst in cancer cells peak accumulation of alkyl-SiNCs occurred within a half hour following the exposure to alkyl-SiNC suspension, the accumulation was considerably slower in the non-malignant cells (Figure 4a). The peak accumulation of the nanocrystals in malignant cells showed some variation in the extent and rate of uptake between the cell types. Nevertheless there was substantial intracellular accumulation, and a high percentage of all malignant cells showed a significant degree of accumulation (Figure 4b–d). There was a time related decrease in fluorescent intensity, indicating that alkyl-SiNC intracellular internalization is followed by ejection, with the peak being reached and then ejection becoming greater than the rate of internalization. In contrast to cancer cells, the uptake in all non-malignant human primary cells was considerably slower, and degree of accumulation and percentage of cells internalizing alkyl-SiNCs was markedly reduced when compared to malignant cells.

**Figure 5:**
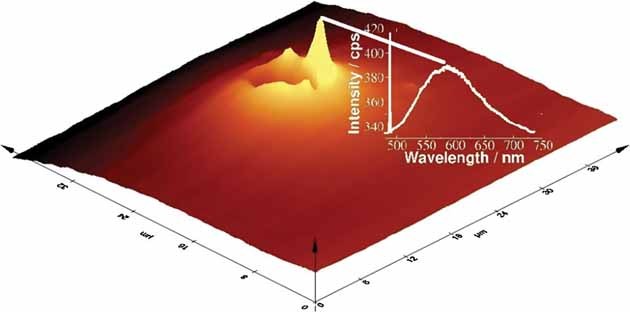
A 3D intracellular luminescence signal from SiNCs within Hela cells. The cells were grown in DMEM then exposed to 2 µL of NCs suspended in DMSO for 60 min. The cells were washed twice with PBS and fixed with a fixative solution of 0.15 m citrate aqueous solution (40%) and acetone (60%) for 30 s. The *λ*_excitation_ was the 488nm line of an argon laser. Each pixel contains a whole fluorescence spectrum over the range of 488 < *λ*/nm < 750. The inset shows one of the spectra that correspond to the peak located in the cell nucleus.

One of the long term objectives of this study is to eventually utilize the NCs, with suitably modified surfaces, to monitor in vivo real-time intracellular signaling pathways and structures, where alkyl-SiNCs with specific active protein domains or oligonucleotides will be used. With this objective in mind, an intracellular localization of alkyl-SiNCs was performed using luminescence microspectroscopy. A 3D intracellular luminescence signal from alkyl-SiNCs suspended in ether was successfully obtained in Hela cells (Figure 5). Our aim is to combine luminescence and signals from proteins or nucleic acid that interact with cognate ligands on NCs to determine spatiotemporal localization together with information about functional interaction. This technique would offer potential application for studying and even manipulating intracellular interaction that are of importance in various pathways involved in regulating a variety of cellular functions.

### Alkyl-SiNC Endocytosis Inhibition

**Figure 6:**
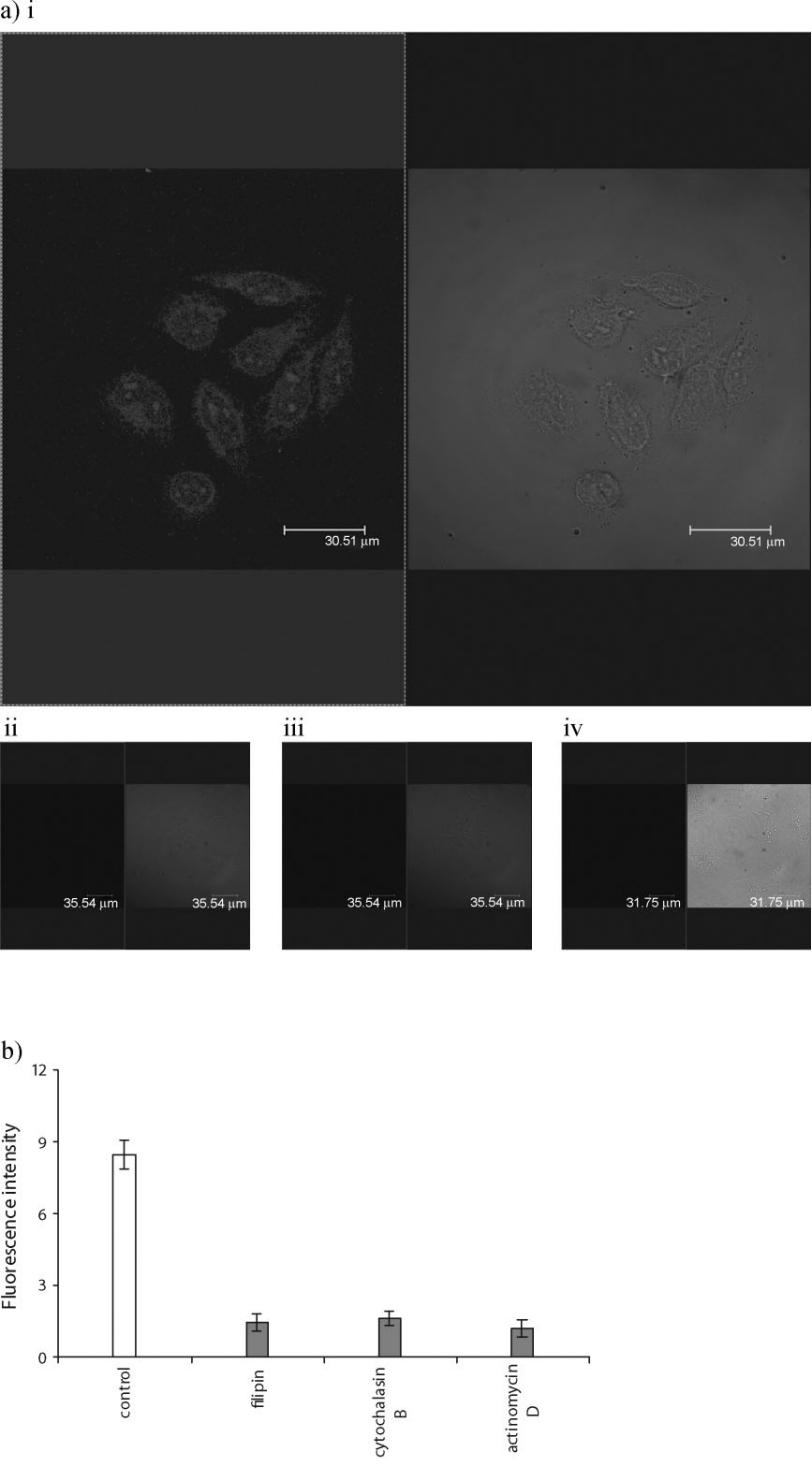
Effect of pinocytosis and motility inhibitors on uptake of alkyl-SiNCs by Hela cells. The pinocytosis inhibitors almost completely blocked internalization of nanocrystals. a) The fluorescence images showing accumulation of alkyl-SiNCs as fluorescence intensities of cells that had been pre-treated for 1 h with (i) vehicle exposed to NCs (white column), (ii) filipin (5 µg mL^−1^), (iii) cytochalasin B (5 µg mL^−1^), and (iv) actinomycin D (10 µm) (dark columns) for 2 h. b) The fluorescent intensities were measured with cells that had been exposed to vehicle (i) and appropriate pinocytosis inhibitors (ii, iii, iv). In order to quantify the extent of intracellular NC accumulation, the NC fluorescence was determined (ranged between 15 to 20 different cells) using the Simulator software.

The precise mechanism of internalization of NCs by cells has not been studied. Therefore, in order to address this, cells in culture were treated with established inhibitors of endocytosis, namely filipin, cytochalasin B, and actinomycin D. These studies were carried out using Hela cells as these are one of the cell-lines that had demonstrated an avid uptake of the alkyl-SiNCs. Incubation of these cells with filipin, a cholesterol binding dye, or cytochalasin B, an inhibitor of microfilaments, led to profound inhibition of intracellular accumulation of alkyl-SiNCs. There was over 80% reduction in alkyl-SiNC-related cell fluorescence in cells treated with these inhibitors (Figure 6). Treatment of cells with actinomycin D (10 µm, 2 h),^19^ which inhibits cell motility, was also associated with almost similar magnitude of inhibition in the intracellular accumulation of alkyl-SiNCs as seen with filipin and cytochalasin B.

## Discussion

Despite rapid and significant intracellular accumulation of alkyl-SiNCs by human cells, exposure of cell-lines to the nanoparticles for up to 24 h was not associated with any demonstrable cell toxicity (Figure 3). The accumulation of alkyl-SiNCs in all cells was found to show a time-dependent change, reaching a peak and followed by a gradual decline, which implies an active mechanism of uptake as well as possible extrusions of the accumulated alkyl-SiNCs from intracellular compartments. The inference of extrusion of the particles, seen in some of the cell-lines, is the most likely explanation since at physiological pH the SiNCs retain about 90% fluorescence intensity after 1 day.^20^ This observed transcytosis, comprising intracellular internalization and ejection of alkyl-SiNCs by established cell-lines of human malignant cells, was relatively efficient, swift, and occurred spontaneously. Alkyl-SiNCs were found distributed within cytosolic and nuclear compartments (Figures 4 and 5). The rate and extent of accumulation of alkyl-SiNCs showed only minor differences between neoplastic cell-lines. There were, however, marked differences between the rate and extent of intracellular accumulation between cancerous and non-cancerous cells. The accumulation of alkyl-SiNCs was demonstrated to be primarily due to endocytosis, as inhibitors of endocytosis and cell motility were found to demonstrate a very significant suppression in the rate uptake and on the extent of intracellular accumulation (Figure 6). In addition, arresting cell cycle by exposure to actinomycin D was also associated with profound inhibition in intracellular internalization, implying that alkyl-SiNC uptake by the cells is largely dependent on internalization of membrane and cell cycle. The uptake was found to be positively correlated with the alkyl-SiNC concentration. Alkyl-SiNCs were internalized within 5 min, and the cellular accumulation increased with incubation time in the presence of nanoparticles in the medium.

The net accumulation of nanoparticles within cells is determined by the rate of endocytosis and exocytosis. The process of endocytosis that involves membrane rafting occurs by membrane internalization, and is known to be affected by a variety of factors. In this regard, such membrane properties as membrane tension, lipid content, and its consequent effect on membrane fluidity have been shown to have a significant effect.^22^,^23^ The role of lipid rafts is of immense interest as these focal membrane regions have been shown to have a critical role in endocytosis as well as a variety of other cellular functions.^24^ The depletion of cholesterol from the membrane leads to inhibition of endocytosis primarily by affecting caveolin-coated pit internalization.^25^,^26^ Therefore, the inhibitory effect of filipin on the internalization of alkyl-SiNCs is interesting as it suggests that endocytosis of these nanoparticles is cholesterol-dependent and involves caveolin-coated rafts. In this context, the extent of inhibition by cytochalasin B is expectedly of similar magnitude as that by filipin, as both inhibit rafting, albeit at different points.

In view of the above considerations, the marked difference between the rate of internalization and net accumulation observed between the normal and neoplastic cells is likely to result from multiple factors. For instance, it has long been recognized that malignancy leads to molecular changes in the plasma membrane that significantly alter biophysical properties.^27^ Indeed, plasma membranes of neoplastic cells have increased fluidity compared to membranes of non-cancerous cells, and plasma membrane fluidity is positively correlated with the capability of cancer cells to form metastases.^27^ It is being increasingly recognized that mobility of molecules within the plasma membrane is dependent on the fluidity of the membrane, that is, a higher fluidity makes it easier for molecules to move and form clusters.

Alkyl-SiNCs showed no evidence of overt or minor toxicity in in vitro studies with a variety of human primary and established cell-lines. This has been evident from the lack of acute effect on the rate of cell morphology, apoptosis, and cell viability. In more chronic studies, which involved exposing murine macrophages RAW 264.7 to Alkyl-SiNCs over 12 days, no significant effect on the cell proliferation was evident. Recent observations in RAW 264.7 and human skin cell-line A431 have also found that alkyl-SiNCs lack cytotoxicity.^28^,^29^ These NCs contain no heavy metals and are capped by a covalently bonded alkyl monolayer that is likely to render them less toxic when compared to nanoparticles incorporating heavy metals, such as cadmium or lead. Concerning nanoparticle toxicity as well basic cell biology, the observation of accumulation of alkyl-SiNCs being followed by a decline is of interest. Vigorous exclusion of alkyl-SiNCs implies an existence of perhaps a protective mechanism involved in actively extruding foreign bodies from the intracellular space. In our study in human cancer cell-lines, expulsion was evident even in the presence of nanoparticles in the medium. This phenomenon may be explained by the fact that most cancer cells have been shown to develop multidrug resistance by virtue of the ability to translocate a wide variety of substrates across extra- and intracellular membranes. The translocation is carried out by the adenosine 5′-triphosphate (ATP)-binding cassette (ABC) transporter superfamily membrane proteins that can translocate a variety of drugs and metabolic products, including lipids and sterols. Over-expression of multidrug resistance protein 1 (MRP1/ABCC1), which belongs to the ABC superfamily of transmembrane proteins, has been demonstrated in cancer cell-lines and tumors that are multidrug resistant. It confers resistance on cancer cells by ATP-dependent efflux of anticancer drugs, and has been demonstrated to transport a wide array of structurally diverse substrates.^30^ From the point of view of NC efflux, it is of interest to note that although the precise mechanism of ABC transportation is unclear, a model known as the hydrophobic vacuum cleaner model states that the transporter interacts with molecules indiscriminately from the lipid phase based on their hydrophobicity.^31^ As has been recently seen, we too observed that internalization into cytosol was followed by relocalization into nuclear and cytoplasmic compartments (Figure 5).^32^

In conclusion, we have found alkyl-capped NCs lack in vitro cellular toxicity and show marked differences in the rate and extent of accumulation between human malignant and normal cells. This uptake of the NCs was primarily observed by cholesterol-dependent endocytosis and an intracellular distribution profile showing widespread cellular distribution. These observations may offer potential for diagnosis and targeted drug delivery for neoplastic conditions.

## Experimental Section

### Preparation of Alkyl-Capped Nanocrystals

The preparation of the alkylated SiNCs used here has been discussed in more detail elsewhere.^18^,^20^,^21^ A brief summary of the procedures is provided. First, photoluminescent porous silicon layers were formed by galvanostatic anodization of p-Si(100) wafer (boron doped, 10 cm resistivity, Compart Technology, Peterborough, UK) in a 1:1 v/v solution of 48% aqueous hydrofluoric acid and ethanol solution. A layer of luminescent porous silicon was made at high current density (5 min at 500 mA cm^−2^) and used directly for formation of C_11_-SiNCs by reflux in undecene/toluene without cleaving the porous layer from the chip. The hydrogen-terminated chip was dried under vacuum on a grease-free vacuum line (employing Young's taps) and then transferred into a Schlenk flask in which the hydrosilation reaction to form C_11_-SiNCs was performed. The porous silicon was refluxed for 4 h in a dry toluene solution (Merck, distilled over Na) containing 1 mol dm^−3^ of the alkene (1-undecene, Merck) until a clear yellow liquid formed. Solvent and unreacted alkene were removed under reduced pressure with a rotary pump to leave a waxy yellow powder of C_11_-SiNCs.

### Nanocrystal Internalization Efficiency

The nanocrystals, being highly lipophilic due to the surface alkyl moiety, were suspended in a range of solvents prior to being introduced in (aqueous) culture medium. The NC solubility was tested in different lipophilic solvents, including THF, toluene, DMSO and ether (Sigma-Aldrich). Following solubilization of the NCs in the respective solvents, cells were exposed for variable times (0.25, 0.5, 1, 2, 4, and 24 h) to different concentrations of the NCs. Toluene and THF, whilst effective as solvents for the NCs, could not be used due to a relatively high degree of toxicity to all cells. The solubility of the NCs was rather reduced in DMSO when compared with other solvents, and also demonstrated relatively diminished uptake by the cells in comparison with the other solvents. Ether was found to be an optimum solvent in terms of solubility and lack of cell toxicity when used 1:500. Therefore, unless stated otherwise, all studies were performed using NCs suspended in ether in a concentration of 1:500. Kinetic experiments were performed to determine the optimum incubation exposure period for NC uptake, maximum uptake, which varied depending on the cell type, was seen to occur between 30 min and 4 h. Therefore, a study aimed to determine the efficiency of uptake was performed at 0.5, 1, 2, and 4 h for Hela cells, and 2 and 4 h for all the other cells. The cells were examined under a confocal microscope (*λ*_excitation_ 488nm and *λ*_emission_ between 550 and 650 nm), and in order to determine the efficiency of uptake, both the number of positive cells that had taken up the NCs and the extent of uptake measured by the fluorescent intensity in cells were determined. The fluorescence was measured against control cells that had been exposed to an appropriate vehicle, comprising solvent only, for the same length of time as the NCs. In order to quantify the amount of fluorescence into the cells, between 5 to 8 different cells were quantified using specific software called Simulator. The baseline was done against an equal number of cells from the vehicle experiment.

### Apoptosis Assay

The apoptosis assays (DeadEnd Colorimetric TUNEL Assay, Promega) were performed accordingly to the manufacturer instructions. Briefly, the cells were first cultured on poly-L-lysine coated microscope slides (Sigma) and incubated with NCs for 30 min and 1 h, respectively. The cells were then washed with phosphate buffer saline (PBS) and fixed with a fixative solution comprised of 0.15 m citrate aqueous solution (40%) and acetone (60%) for 30 s. Biotinylated nucleotide is incorporated at the 3′-OH DNA ends using the recombinant terminal deoxynucleotidyl transferase, (rTdT). Horseradish peroxidase-labeled streptavidin is then bound to the biotinylated nucleotides, which are detected using the peroxidase substrate, hydrogen peroxide, and the stable chromogen, diaminobenzidine. Apoptotic nuclei are stained dark brown. A positive control was performed by the addition of DNase1 for 10 min to the cells as it results in fragmentation of the chromosal DNA and a negative control was realized with cells in absence of the enzyme rTdT.

### Cell Viability Assay

Hela growing in a 75 cm^2^ cell-culture flask (Corning, UK) were harvested by trypsinization (0.05% trypsin, 0.02% ethylenediaminetetraacetic acid (EDTA)) and equal volumes of cells were aliquoted into 24-well cell-culture plates (Corning) containing Dulbecco's modified essential medium (DMEM) supplemented with 10% fetal calf serum (FCS). The cells were incubated at 37 ° for 24 h to allow cell attachment and were prepared for experiments by overnight serum starvation in medium containing 0.5% FCS (1 mL). Thereafter, cells were exposed to either NCs suspended in 2 µL ether (Sigma) and controls were exposed to vehicle (2 µL ether) alone. All experiments (*n* = 6) were carried out for 1, 4, and 24 h and experiments were repeated on at least three different occasions. Cell wells containing 1 mL of DMEM supplemented with 10% FCS alone served as control experiments. The control and test plates were then treated and analyzed under identical conditions, allowing paired comparison of the corresponding wells. At the end point of each exposure time (1, 4, or 24 h), cell viability was estimated using CellTiter-Blue Cell Viability Assay (Promega) following manufacturer's protocol. Briefly, 200 µL of CellTiter-Blue reagent (1:5 v/v) was added to each well containing 1 mL of medium, shaken for 10 s and incubated at 37 °C for 4 h. At the end of each incubation, the cell-wells were shaken for a further 10 s and fluorescence was measured on a Cytofluor 2300 fluorimeter (Millipore, UK) with the filter set 1 giving an excitation wavelength of 579 nm and emission of 584 nm.

### Cell Proliferation Assay

Hela cells growing in a 75 cm^2^ cell-culture flask (Corning, UK) were harvested by trypsinization (0.05% trypsin, 0.02% EDTA) and equal volume of cells were aliquoted into 24-well cell-culture plates (Corning) containing DMEM supplemented with 10% FCS. The cells were incubated at 37 ° for 24 h to allow cell attachment and were prepared for experiments by overnight serum starvation in medium containing 0.5% FCS (1 mL). Thereafter, cells were exposed to NCs suspended in 2 µL ether (Sigma) and controls were exposed to vehicle (2 µL ether) alone. All experiments (*n* = 8) were carried out for 2, 4, and 24 h and experiments were repeated on at least three different occasions. Cell wells containing 1 mL of DMEM medium supplemented with 10% FCS alone served as control experiments. The control and test plates were then treated and analyzed under identical conditions allowing paired comparison of the corresponding wells. At the end point of each exposure time (2, 4, or 24 h), cell viability was estimated using cell proliferation assay by MTT (3-(4,5-dimethylthiazol-2-yl)-2,5-diphenyltetrazolium bromide) (Promega) following manufacturer's protocol. Briefly, serum-starved cells exposed to NCs or vehicles were incubated with MTT solution (1 mg mL^−1^) for 2 h in CO_2_ incubator 5%. The MTT solution was discarded and acidic isopropanol was added to the cells and shaken for 30 min to allow complete solubilization. Absorbance was read at 560 nm, blanking on control wells.

### Pinocytosis and Motility Inhibition Studies

The pinocytosis and motility inhibitors used were filipin (5 µg mL^−1^) and cytochalasin B (5 µg mL^−1^). These studies were carried out on Hela cells that had been cultured on glass cover slips in DMEM culture medium as described above. The cells were incubated for 1 h with filipin or cytochalasin B at (5 µg ml^−1^) before NCs or vehicle was added. After further incubation of 1h at 37 °C, 5% CO_2_, the cells were washed and then fixed and examined under a confocal microscope. In separate experiments Hela cells were grown on glass coverslips and treated with actinomycin D (10 µm) for 2h and then exposed to NCs. This dose of actinomycin is known to inhibit transcription and lead to cessation of the cell cycle.

### Confocal Laser Scanning Microscopy

The LEICA TCS SP2 system with a LEICA DM IRE2 microscope having an Argon/Krypton Laser (Leica TCS SP2, Spectral Confocal and Multiphoton Microscope, Leica Microsystems Ltd., Milton Keynes, UK) was used in these experiments. This system allows the imaging of a single focal plane as well as a series of planes—horizontal or vertical. The microscope is a no-compromise true point-scanning system with extremely high sensitivity and theoretical maximum x-, y- and z-resolution. Observations were made using a HCX PLAPOCS 40.0 × 1.25 oil immersion objective lens and a stepper motor was used for collecting Z-series images.

A CRM200 confocal Raman microscope (Witec GmbH, Ulm, Germany) was used to obtain luminescence spectral images. The 488 nm line of an argon ion laser provided the excitation light and the emitted and/or scattered light passed through a Raman edge filter to remove elastically scattered light. The filtered light was collected by a multimode optical fiber that also served as the confocal pinhole. The lateral spatial resolution of the instrument is close to the diffraction limit, that is, about 250 nm. The collected light was analyzed by a spectrograph with typical settings of 150 lines mm^−1^ (grating), an integration time of 0.1 s per pixel, and 256 × 256 pixels for each image.
